# Survival rates and prognostic factors in right- and left-sided colon cancer stage I–IV: an unselected retrospective single-center trial

**DOI:** 10.1007/s00384-021-04005-6

**Published:** 2021-08-26

**Authors:** Claudius E. Degro, Richard Strozynski, Florian N. Loch, Christian Schineis, Fiona Speichinger, Lucas D. Lee, Georgios A. Margonis, Johannes C. Lauscher, Katharina Beyer, Martin E. Kreis, Carsten Kamphues

**Affiliations:** 1 Department of General, Visceral and Vascular Surgery, Charité – Universitätsmedizin Berlin, corporate member of Freie Universität Berlin and Humboldt-Universität zu Berlin, Campus Benjamin Franklin, Hindenburgdamm 30, 12203 Berlin, Germany; 2 Department of Surgery, Memorial Sloan Kettering Cancer Center, 1275 York Avenue, 10065 New York, NY USA

**Keywords:** Colorectal cancer, Laterality, Survival, Mortality predictors

## Abstract

**Purpose:**

Colorectal cancer revealed over the last decades a remarkable shift with an increasing proportion of a right- compared to a left-sided tumor location. In the current study, we aimed to disclose clinicopathological differences between right- and left-sided colon cancer (rCC and lCC) with respect to mortality and outcome predictors.

**Methods:**

In total, 417 patients with colon cancer stage I–IV were analyzed in the present retrospective single-center study. Survival rates were assessed using the Kaplan–Meier method and uni/multivariate analyses were performed with a Cox proportional hazards regression model.

**Results:**

Our study showed no significant difference of the overall survival between rCC and lCC stage I–IV (*p* = 0.354). Multivariate analysis revealed in the rCC cohort the worst outcome for ASA (American Society of Anesthesiologists) score IV patients (hazard ratio [HR]: 16.0; CI 95%: 2.1–123.5), CEA (carcinoembryonic antigen) blood level > 100 µg/l (HR: 3.3; CI 95%: 1.2–9.0), increased lymph node ratio of 0.6–1.0 (HR: 5.3; CI 95%: 1.7–16.1), and grade 4 tumors (G4) (HR: 120.6; CI 95%: 6.7–2179.6) whereas in the lCC population, ASA score IV (HR: 8.9; CI 95%: 0.9–91.9), CEA blood level 20.1–100 µg/l (HR: 5.4; CI 95%: 2.4–12.4), conversion to laparotomy (HR: 14.1; CI 95%: 4.0–49.0), and severe surgical complications (Clavien-Dindo III–IV) (HR: 2.9; CI 95%: 1.5–5.5) were identified as predictors of a diminished overall survival.

**Conclusion:**

Laterality disclosed no significant effect on the overall prognosis of colon cancer patients. However, group differences and distinct survival predictors could be identified in rCC and lCC patients.

## Introduction

Colorectal cancer (CRC) is one of the world’s foremost diagnosed cancer types and a leading cause of death in western countries. According to the GLOBOCAN database, CRC counted in 2018 1.8 million new cases and caused 860.000 deaths worldwide [1]. The tumor location of CRC varies within the large intestine, but shows a clear abundance in the rectosigmoid compared to other colonic segments [2]. Nevertheless, these differences seemed to alter over the last decades with an increase of carcinoma diagnosed in the ascending colon which display specific sex, age, and biological properties [3, 4]. Apart from surgical resection, targeted strategies and determination of relevant prognostic factors seemed to gain crucial relevance in the multidisciplinary treatment of CRC [5–7]. However, the debatable aspect of different tumor entities defined by their proximal or distal location referring to functional implications in terms of personalized therapeutic options and predictive factors remained widely unsolved. Previous studies have yet disclosed molecular and chromosomal differences between left-sided and right-sided colon cancer (lCC and rCC) what could likely correlate with their embryogenetic origin [8, 9]. Indeed, those segments including the distal third of the transverse colon, the splenic flexure, the descending colon, the sigmoid colon, and the rectum derive from the hindgut whereas the remaining colon develops from the midgut hence displaying potential differences in carcinogenesis and tumor progression, whether in an incomprehensive manner [10]. Further studies, analyzing clinical and prognostic factors of rCC and lCC, could reveal feasible differences among these potential distinct tumor entities albeit with diverging results [11–13]. Several surveys already demonstrated a worse overall survival of rCC compared to a left-sided tumor location [14, 15]. Surprisingly, a recent study from Warschkow and colleagues 2016 [16] revealed a superior overall and cancer-specific survival of rCC stage I–III. Beyond that, Ishihara and colleagues [17] demonstrated 2018 in a multicenter study a predominant relapse-free survival of rCC, at least for stage II–III. However, most of these studies were characterized by a strictly defined patient population based on the tumor stage and lack an unselected sample of those colon cancer (CC) patients that can be found as cross-section at most clinical institutions.

The present study aims to address these circumstances and represents a retrospective single-center study of CC patients’ stage I–IV to detect clinicopathological differences between rCC and lCC with special regard to the overall survival rate and prognostic factors. A total of 417 patients that were diagnosed with and treated for CC of any stage between February 2009 and May 2019 at the Charité University Hospital Berlin, Campus Benjamin Franklin, Berlin, Germany, were acquired and analyzed. Laterality was determined by the vascular supply from either the superior mesenteric artery (SMA) or inferior mesenteric artery (IMA) matching the embryogenetic definition. Patients with rectal cancer were discarded due to its unique anatomical characteristics (i.e., hematogenous metastasis via inferior mesenteric vein and internal iliac vein) and especially various treatment strategies (i.e., neoadjuvant chemotherapy, radiotherapy).

## Methods

### Patients

The current study represents a retrospective single-center study that constitutes a total number of 417 patients with histologically confirmed CC who underwent a curative intended surgical resection between February 2009 and May 2019 at the Charité University Hospital Berlin, Campus Benjamin Franklin, Berlin, Germany. Study observations were performed in accordance with local ethical committees (EA2/208/19). To reproduce an unselected population, present at most surgical centers, patients with a tumor location in the colon and the rectosigmoid were included regardless of their tumor stage. However, due to a different tumorigenesis or specific therapeutic approaches, patients with the following characteristics were discarded from further study investigations: isolated rectal cancer (tumor location ≤ 16 cm from the anal verge, [18]), synchronous carcinoma, primary palliative therapy, and IBD (inflammatory bowel disease)-associated CC. Patient characteristics and disease-related parameters were extracted online from the local institutional database and analyzed offline in a retrospective manner. Follow-up data were obtained from either the abovementioned local data files or the Charité Comprehensive Cancer Center database, if follow-up care was proceeded elsewhere to reduce the number of dropouts.

### Definition of laterality and colic flexures

Laterality was primary determined on the basis of the embryogenetic development of the intestinal tract and its blood supply [19]. In particular, a tumor location within the cecum, the ascending colon, the right colic flexure, and the subsequent two-thirds of the transverse colon were defined as rCC (midgut, SMA region) whereas a tumor that was diagnosed in the distal third of the transverse colon, the left colic flexure, the descending colon, and the (recto)sigmoid colon was determined as lCC (hindgut, IMA region). However, due to various anastomoses between SMA and IMA especially in the area of the transverse colon, the middle colic artery was used as a radiographic and intraoperative reference to subdivide the transverse colon into a distal part (aboral the middle colic artery) and proximal/mid part (oral the middle colic artery). According to the current literature [20], the left colic flexure comprised the distal third of the transverse colon and the proximal descending colon. Analog to this definition, we defined the right colic flexure as the proximal third of the transverse colon together with the distal ascending colon.

### Surgical procedures and indications of adjuvant chemotherapy

In our study cohort, laparoscopy was the surgical procedure of choice. Laparotomy was performed when laparoscopy was either not achievable or contraindicated (i.e., patients with extensive surgery in the past, vast infiltrating tumors, pronounced comorbidities). Conversion was reserved to control severe intraoperative complications and to improve the view on the surgical field if the laparoscopic procedure failed to ensure ideal operative conditions. The extent of mesocolic resection comprised a complete or partial (D2 lymphadenectomy) mesocolic excision in left and right hemicolectomy under preservation of the greater omentum (except for tumor penetration or adherence). In extended right hemicolectomy, generally performed for tumors of the right colic flexure and the mid transverse colon (oral the middle colic artery), mesenterectomy also included a partial resection of the greater omentum involving lymph nodes alongside the greater curvature and the head of the pancreas. Likewise, extended left hemicolectomy was performed for tumors of the left colic flexure including the distal part of the transverse colon (aboral the middle colic artery) and consisted of a partial omental resection with removal of lymph nodes along the tail of the pancreas. An exclusive transverse colectomy was not performed in our study cohort. Patients with metastatic disease (stage IV) received an additional concomitant or sequential resection of their organ metastases.

Additive to the surgical treatment, adjuvant chemotherapy was regularly recommended to stage III patients. In stage II patients, adjuvant chemotherapy was applied contingent on cancer-specific risk factors (i.e., pT4 tumors, tumor perforation, reduced lymphadenectomy), according to current national guidelines. In stage IV patients, additive treatments were defined individually in local tumor boards and included perioperative and postoperative (adjuvant) chemotherapy strategies.

### rCC and lCC analysis

To reveal potential effects of the primary tumor location on survival rates and detect specific prognostic factors, the study population was further segregated into rCC and lCC subgroups depending on their vascular supply from either the SMA or IMA. Patient- and disease-related parameters (demographics, histopathology, molecular pathology, tumor marker, operative details, surgical complications [21], follow-up) were analyzed in the entire population and the two subgroups separately to disclose clinicopathological differences.

### Statistics

Statistical analysis was performed with IBM SPSS Statistics 27.0 (IBM Corp. Released 2020. IBM SPSS Statistics for Windows, Version 27.0. Armonk, NY: IBM Corp). Survival rates were assessed using the Kaplan–Meier method and compared with the log-rank test. Covariates with a *p*-value < 0.05 in the Kaplan–Meier analysis were subsequently applied to an univariate and multivariate (with backward elimination) Cox proportional hazards regression model. Group-specific differences were analyzed with either the unpaired *t-*test or the *χ*^2^-test respectively. Continuous variables are shown as mean ± SEM or as median with range (min.–max.) throughout. A *p*-value < 0.05 was assigned to be statistical significant.

## Results

### Entire CC cohort

Based on the institutional database, we identified a total number of 417 patients that underwent an oncological colon resection between February 2009 and May 2019. The mean follow-up period was 33.1 ± 1.4 months with a median of 25.3 months (0.1–118.9 months). The mean age of the cohort was 66.9 ± 0.7 years with a median of 70 years (17–98 years) and included 247 (59.2%) male and 170 (40.8%) female patients. rCC was found in 230 (55.2%) and lCC in 187 (44.8%) patients. The two-year (2-y) and five-year (5-y) overall survival rates were 75.0% and 62.0% respectively. The mean overall survival was 75.5 ± 3.0 months.

A total of 116 patients (29.6%) received a complete mesocolic excision and 276 patients (70.4%) a partial mesocolic excision (D2 lymphadenectomy). In the stage IV subgroup, 31 patients (63.3%) received concomitant and 18 patients (36.7%) sequential resection of their organ metastases (with 44.4% preceding and 55.6% subsequent metastasectomies). Adjuvant chemotherapy was performed in 139 patients (33.3%) including 62 stage IV (44.6%), 63 stage III (45.3%), and 14 stage II (10.1%) patients. In the CC stage I–III subpopulation (*n* = 330), 40 patients (12.1%) revealed a recurrence after a mean time of 20.1 ± 2.3 months (median: 16.7 months, range: 1.7–63.8 months) with 30.0% of all recurrences restricted to liver metastases. A detailed overview of patient- and disease-related parameters is shown in Table 1.

**Table 1:** Patient characteristics and disease-related parameters

CC (*n* = 417)	
Age (years)	
< 65	157 (37.6%)
≥ 65	260 (62.4%)
Mean	66.9 ± 0.7
Sex	
Female	170 (40.8%)
Male	247 (59.2%)
ASA-score	
I	37 (8.9%)
II	225 (54.0%)
III	150 (36.0%)
IV	5 (1.2%)
BMI (kg*m^−2^)	
≤ 18.4	14 (3.4%)
18.5–24.9	187 (44.8%)
25.0–29.9	141 (33.8%)
≥ 30	75 (18.0%)
Mean	26.2 ± 0.3
CEA (µg/l)*^1^	
0–5.0	274 (66.8%)
5.1–20.0	82 (20.0%)
20.1–100.0	38 (9.3%)
> 100.0	16 (3.9%)
Mean	62.3 ± 22.1
Tumor location	
rCC	230 (55.2%)
lCC	187 (44.8%)
Mesenterectomy*^2^	
Complete mesocolic excision	116 (29.6%)
Partial mesocolic excision (D2)	276 (70.4%)
Tumor entity	
NST	184 (44.1%)
Tubular	152 (36.5%)
Mucinous	66 (15.8%)
Others	15 (3.6%)
pT-stage	
Tis/T1	48 (11.5%)
T2	76 (18.2%)
T3	216 (51.8%)
T4a/b	77 (18.5%)
pN-stage*^3^	
N0	239 (57.5%)
N1a/ N1b/N1c	38 (9.1%)/51 (12.3%)/26 (6.3%)
N2a/N2b	26 (6.3%)/36 (8.7%)
pM-stage	
M0	330 (79.1%)
M1a/ M1b/ M1c	66 (15.8%)/12 (2.9%)/9 (2.2%)
Metastasectomy*^4^	
Concomitant	31 (63.3%)
Sequential	18 (36.7%)
Lymph node ratio*^5^	
0.0	259 (62.6%)
0.01–0.29	106 (25.6%)
0.30–0.59	32 (7.7%)
0.60–1.0	17 (4.1%)
Grade*^6^	
G1	20 (4.8%)
G2	288 (69.4%)
G3	106 (25.5%)
G4	1 (0.2%)
Lymphangioinvasion	
L0	315 (75.5%)
L1	102 (24.5%)
Venous invasion	
V0	362 (86.8%)
V1	55 (13.2%)
Perineural invasion	
Pn0	408 (97.8%)
Pn1	9 (2.2%)
R-stage	
R0	403 (96.6%)
R1	14 (3.4%)
Microsatellite instability*^7^	
MSS	231 (84.3%)
MSI	43 (15.7%)
Type of operation	
Laparoscopy	156 (37.4%)
Laparotomy	226 (54.2%)
Conversion	35 (8.4%)
Clavien-Dindo*^8^	
0–II	285 (70.0%)
III–IV	122 (30.0%)
Chemotherapy (adjuvant)	139 (33.3%)
Recurrence (stage I–III)*^9^	
Yes	40 (12.1%)
No	290 (87.9%)
Site of recurrence	
Local	9 (22.5%)
Peritoneal	10 (25.0%)
Hepatic	12 (30.0%)
Others	9 (22.5%)
Time to recurrence (months)	20.1 ± 2.3
Overall survival (months)	75.5 ± 3.0
2-year-overall survival (%)	75.0
5-year-overall survival (%)	62.0

*CC* colon cancer, *ASA* American Society of Anesthesiologists, *BMI* body mass index, *CEA* carcinoembryonic antigen, *rCC* right-sided colon cancer, *lCC* left-sided colon cancer, *D2* D2 lymphadenectomy, *NST* no special type, *MSS* microsatellite stable, *MSI* microsatellite instable, *Clavien-Dindo* Clavien-Dindo classification of surgical complications

*^1^*n* = 410; *^2^*n* = 392; *^3^*n* = 416; *^4^*n *= 49; *^5^*n* = 414; *^6^*n* = 415; *^7^*n* = 274; *^8^*n* = 407; *^9^*n* = 330

### Survival rates of rCC and lCC

**Fig. 1:**
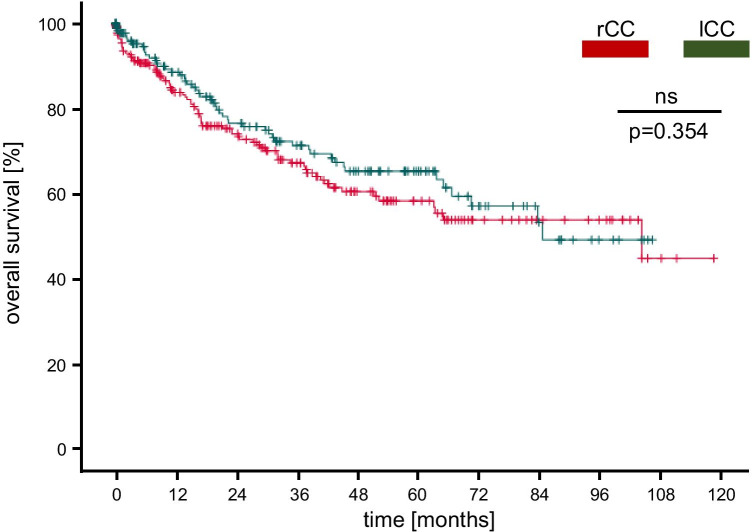
Overall survival of rCC and lCC stage I–IV: Overall survival of rCC and lCC patients revealed no significant difference (p = 0.354). Censored events are represented as vertical lines within the Kaplan–Meier graph. rCC, right-sided colon cancer; lCC, left-sided colon cancer

**Fig. 2:**
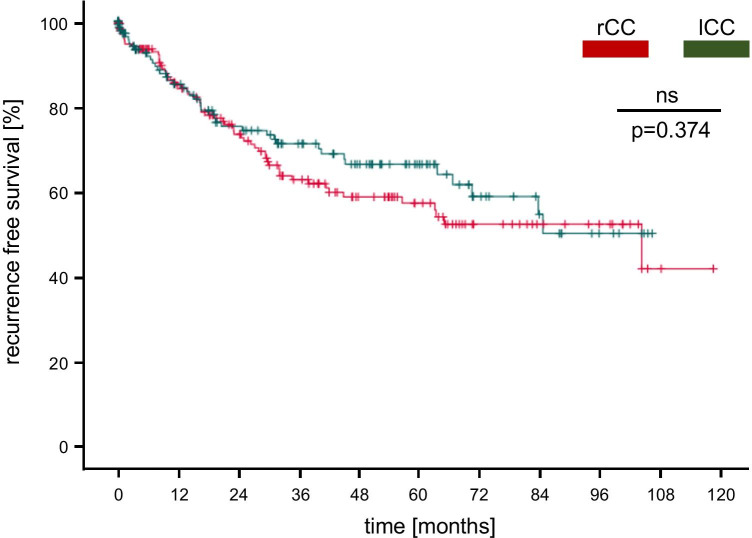
Recurrence-free survival of rCC and lCC stage I–III: Recurrence-free survival of rCC and lCC patients revealed no significant difference (p = 0.374). Censored events are represented as vertical lines within the Kaplan–Meier graph. rCC, right-sided colon cancer; lCC, left-sided colon cancer

To detect potential differences between rCC and lCC survival rates, endorsing the hypothesis of diverse tumor entities, the population was segregated based on its tumor location and analyzed separately. Our study showed no significant difference of the overall survival rate between rCC and lCC stage I–IV (Fig. 1, *p* = 0.354). The 2-y and 5-y overall survival rates varied between 73.0 and 58.0% for rCC and 77.0 and 65.0% for lCC. To determine whether the recurrence-free survival differed depending on the tumor location, we analyzed patients with CC stage I–III (*n* = 330) and revealed likewise no difference between the rCC and lCC cohort (Fig. 2*;* rCC: 2-y/5-y: 73.0%/58.0% vs. lCC: 2-y/5-y: 75.0%/ 67.0%, *p* = 0.374).

### Group differences of rCC and lCC

Analyzing group-specific factors disclosed some distinct differences between rCC and lCC patients (Table 2). Colon carcinoma located in the right colon appeared more frequently in female patients compared to the lCC subpopulation (48.7% vs. 31.0%), whereas men developed predominantly left-sided tumors (69.0% vs. 51.3%, *p* < 0.001). Moreover, rCC showed a higher proportion of a mucinous histological characteristic (19.1% vs. 11.8%) while lCC displayed more tubular carcinoma than rCC (40.6% vs. 33.0%, *p* = 0.027). Further parameters with a significant difference among these two subpopulations were the pN-stage (*p* = 0.012) and the histological grade (*p* = 0.043) that were both more advanced in the rCC group. Furthermore, rCC excelled in their microsatellite instability compared to left-sided tumors (23.9% vs. 5.0%, *p* < 0.001). Besides, no significant difference of the pT-stage was detected (*p* = 0.065). Likewise, rCC and lCC patients showed no difference in the extent of mesenterectomy with 31.3% receiving a complete mesocolic excision in the rCC cohort vs. 27.4% in the lCC cohort (*p* = 0.665). Similar results for both groups were also obtained for the total number and distribution of adjuvant chemotherapies (34.3% vs. 32.1%, *p* = 0.626; rCC: stage IV: 46.8%, stage III: 44.3%, stage II: 8.9%; lCC: stage IV: 41.7%, stage III: 46.7%, stage II: 11.6%, *p* = 0.774) and the type of metastasectomy in stage IV patients (concomitant vs. sequential, *p* = 0.520; rCC: 70.4% concomitant and 29.6% sequential resection with 50.0% preceding and 50.0% subsequent metastasectomies; lCC: 54.5% concomitant and 45.5% sequential resection with 40.0% preceding and 60.0% subsequent metastasectomies). Finally, analyzing the recurrence rate in stage I–III patients revealed no difference of the total number of recurrences (rCC: 13.3% vs. lCC: 10.7%, *p* = 0.460) and the mean time until a recurrence occurred (rCC: 19.6 ± 2.5 months vs. lCC: 20.8 ± 4.4 months, *p* = 0.381). Although showing no statistical significant difference in the site of recurrence (*p* = 0.06), there was a clear tendency for a higher rate of local recurrences in the lCC cohort (43.8% vs. 8.3%) whereas in the rCC cohort, metastases were more frequently found as peritoneal lesions (29.2% vs. 18.7%) and in distant organs (other than the liver, colon, and peritoneum: 33.3% vs. 6.3%). The frequency of hepatic lesions was similar in both groups (rCC: 29.2% vs. lCC: 31.2%).

**Table 2:** Survival analysis and group differences of rCC and lCC

	rCC (*n* = 230)	*p*-value (log-rank)	lCC (*n* = 187)	*p*-value (log-rank)	*p*-value (*χ*^2^/*t*-test)
Age (years)		0.286		< 0.001	0.256
< 65	81 (35.2%)		76 (40.6%)		
≥ 65	149 (64.8%)		111 (59.4%)		
Mean	68.0 ± 0.8		65.6 ± 1.0		0.066
Sex		0.359		0.003	* < 0.001*
Female	112 (48.7%)		58 (31.0%)		
Male	118 (51.3%)		129 (69.0%)		
ASA-Score		< 0.001		< 0.001	0.138
I	14 (6.1%)		23 (12.3%)		
II	128 (55.7%)		97 (51.9%)		
III	86 (37.4%)		64 (34.2%)		
IV	2 (0.9%)		3 (1.6%)		
BMI (kg*m^−2^)		0.313		0.748	0.318
≤ 18.4	11 (4.8%)		3 (1.6%)		
18.5–24.9	99 (43.0%)		88 (47.1%)		
25.0–29.9	79 (34.3%)		62 (33.2%)		
≥ 30	41 (17.8%)		34 (18.2%)		
Mean	26.2 ± 0.4		26.2 ± 0.3		0.977
CEA (µg/l)*^1^		< 0.001		* < 0.001*	0.985
0–5.0	153 (67.4%)		121 (66.1%)		
5.1–20.0	45 (19.8%)		37 (20.2%)		
20.1–100.0	20 (8.8%)		18 (9.8%)		
> 100.0	9 (4.0%)		7 (3.8%)		
Mean	56.6 ± 26.5		69.4 ± 37.2		0.775
Tumor entity		0.987		0.054	0.027
NST	98 (42.6%)		86 (46.0%)		
Tubular	76 (33.0%)		76 (40.6%)		
Mucinous	44 (19.1%)		22 (11.8%)		
Others	12 (5.2%)		3 (1.6%)		
pT-stage		< 0.001		0.014	0.065
Tis/T1	25 (10.9%)		23 (12.3%)		
T2	46 (20.0%)		30 (16.0%)		
T3	108 (47.0%)		108 (57.8%)		
T4a/b	51 (22.2%)		26 (13.9%)		
pN-stage*^2^		< 0.001		0.175	0.012
N0	131 (57.2%)		108 (57.8%)		
N1a/ N1b/N1c	27 (11.8%)/26 (11.4%)/7 (3.1%)		11 (5.9%)/25 (13.4%)/19 (10.2%)		
N2a/N2b	14 (6.1%)/24 (10.5%)		12 (6.4%)/12 (6.4%)		
pM-stage		< 0.001		*< 0.001*	0.579
M0	180 (78.3%)		150 (80.2%)		
M1a/M1b/M1c	36 (15.7%)/7 (3.0%)/7 (3.0%)		30 (16.0%)/5 (2.7%)/2 (1.1%)		
Lymph node ratio*^3^		< 0.001		0.054	0.726
0.0	138 (60.5%)		121 (65.1%)		
0.01–0.29	60 (26.3%)		46 (24.7%)		
0.30–0.59	19 (8.3%)		13 (7.0%)		
0.60–1.0	11 (4.8%)		6 (3.2%)		
Grade*^4^		0.001		0.026	0.043
G1	13 (5.7%)		7 (3.8%)		
G2	146 (63.8%)		142 (76.3%)		
G3	69 (30.1%)		37 (19.9%)		
G4	1 (0.4%)				
Lymphangioinvasion		0.004		*0.393*	0.690
*L0*	172 (74.8%)		143 (76.5%)		
*L1*	58 (25.2%)		44 (23.5%)		
Venous invasion		0.001		0.001	0.207
*V0*	204 (88.7%)		158 (84.5%)		
*V1*	26 (11.3%)		29 (15.5%)		
Perineural invasion		0.083		0.526	0.981
*Pn0*	225 (97.8%)		183 (97.9%)		
*Pn1*	5 (2.2%)		4 (2.1%)		
R-stage		0.064		0.006	0.485
R0	221 (96.1%)		182 (97.3%)		
R1	9 (3.9%)		5 (2.7%)		
Microsatellite instability*^5^		0.449		0.724	< 0.001
MSS	118 (76.1%)		113 (95.0%)		
MSI	37 (23.9%)		6 (5.0%)		
Type of operation		0.091		< 0.001	0.140
Laparoscopy	79 (34.3%)		77 (41.2%)		
Laparotomy	127 (55.2%)		99 (52.9%)		
Conversion	24 (10.4%)		11 (5.9%)		
Clavien-Dindo*^6^		0.113		0.001	0.687
0–II	155 (69.2%)		130 (71.0%)		
III–IV	69 (30.8%)		53 (29.0%)		
Chemotherapy (adjuvant)	79 (34.3%)	0.394	60 (32.1%)	0.908	0.626

*rCC* right-sided colon cancer, *lCC* left-sided colon cancer, *ASA* American Society of Anesthesiologists, *BMI* body mass index, *CEA* carcinoembryonic antigen, *NST* no special type, *MSS* microsatellite stable, *MSI* microsatellite instable, *Clavien-Dindo* Clavien-Dindo classification of surgical complications

^*^^1^n = 227 (rCC), n = 183 (lCC); *^2^n = 229 (rCC); *^3^n = 228 (rCC), n = 186 (lCC); *^4^n = 229 (rCC), n = 186 (lCC); *^5^n = 155 (rCC), n = 119 (lCC); *^6^n = 224 (rCC), n = 183 (lCC)

### Outcome predictors of rCC and lCC

#### Univariate analysis

To further verify distinct parameters that potentially interfere with the overall survival by acting as survival predictors of rCC and lCC, an univariate analysis following the Kaplan–Meier method was used in both groups for each parameter separately (Table 2). Parameters with a significant impact on the overall survival were subsequently applied to an univariate Cox proportional hazards regression model to calculate the individual hazard ratios (HR) (Table 3). In the lCC cohort, an age ≥ 65 years (HR: 3.7; CI 95%: 1.7–7.9, *p* = 0.001) and male sex (HR: 2.8; CI 95%: 1.4–5.9, *p* = 0.005) were associated with an increased mortality risk whereas no significant correlation could be tested for the same characteristics in the rCC group. Beyond that, an advanced pT-stage (T4a/b: HR: 4.6; CI 95%: 1.6–13.4, *p* = 0.005 in rCC. T4a/b: HR: 12.8; CI 95%: 1.7–97.5, *p* = 0.014 in lCC) correlated with a worse overall survival in both groups. Further parameters with a significantly increased hazard in the lCC group were the R-status (R1: HR: 4.6; CI: 95%: 1.4–15.2, *p* = 0.012), as well as the surgical procedure (laparotomy: HR: 2.7; CI 95%: 1.3–5.7, *p* = 0.008; conversion to laparotomy: HR: 13.8; CI 95%: 4.8–40.0, *p* < 0.001) and the severity of complications (Clavien-Dindo III–IV: HR: 2.5; CI 95%: 1.4–4.3, *p* = 0.002). Increased lymph node ratio of 0.6–1.0 (HR: 6.4; CI 95%: 2.8–14.7, *p* < 0.001) and lymphangioinvasion (HR: 2.0; CI 95%: 1.2–3.2, *p* = 0.005) represented exclusive survival predictors in the rCC cohort, while advanced ASA (American Society of Anesthesiologists) score, elevated CEA (carcinoembryonic antigen) blood levels, poor histological grade, and venous invasion were found to reduce the overall survival in both groups (Table 3).

**Table 3:** Univariate analysis of survival predictors of rCC and lCC

	rCC			lCC		
	Hazard-ratio	CI 95%	*p*-value	Hazard-ratio	CI 95%	*p*-value
Age (years)						
< 65	1.0			1.0		
≥ 65	1.3	0.8–2.2	0.288	3.7	1.7–7.9	0.001
Sex						
Female	1.0			1.0		
Male	0.8	0.5–1.3	0.360	2.8	1.4–5.9	0.005
ASA-Score			< 0.001			< 0.001
I	1.0			1.0		
II	1.7	0.4–7.2	0.458	2.3	0.5–10.0	0.256
III	4.7	1.2–19.7	0.032	6.7	1.6–28.1	0.010
IV	12.4	1.7–88.0	0.012	8.1	1.3–48.8	0.023
CEA (µg/l)			< 0.001			< 0.001
0–5.0	1.0			1.0		
5.1–20.0	2.5	1.4–4.3	0.002	2.5	1.2–5.2	0.011
20.1–100.0	4.3	2.2–8.3	< 0.001	6.2	3.0–12.8	< 0.001
> 100.0	4.5	1.7–11.4	0.002	6.0	2.2–16.0	< 0.001
pT-stage			0.001			0.037
Tis/T1	1.0			1.0		
T2	1.4	0.4–4.5	0.568	7.0	0.9–54.9	0.066
T3	2.2	0.8–6.3	0.132	6.6	0.9–48.6	0.064
T4a/b	4.6	1.6–13.4	0.005	12.8	1.7–97.5	0.014
pN-stage			< 0.001			0.604
N0	1.0			1.0		
N1a	1.9	0.9–4.1	0.086			
N1b	2.3	1.1–4.8	0.023	1.0	0.4–2.2	0.955
N1c	2.0	0.5–8.5	0.337	1.3	0.5–3.0	0.617
N2a	3.9	1.8–8.2	< 0.001	2.1	0.9–5.1	0.098
N2b	4.8	2.5–9.1	< 0.001	1.7	0.6–4.3	0.296
pM-stage			< 0.001			< 0.001
M0	1.0			1.0		
M1a	2.3	1.3–4.0	0.005	2.1	1.1–4.1	0.035
M1b	7.1	3.2–15.9	< 0.001	6.6	1.9–22.7	0.003
M1c	3.2	1.0–10.5	0.053	12.4	2.8–54.4	0.001
Lymph node ratio			< 0.001			0.076
0.0	1.0			1.0		
0.01–0.29	2.6	1.5–4.4	< 0.001	0.8	0.4–1.6	0.514
0.30–0.59	4.6	2.2–9.4	< 0.001	1.5	0.6–3.6	0.350
0.60–1.0	6.4	2.8–14.7	< 0.001	3.4	1.2–9.6	0.024
Grade			0.008			0.031
G1	1.0			1.0		
G2	3.2	0.4–23.1	0.256	1.2	0.2–8.9	0.851
G3	5.1	0.7–37.1	0.112	2.6	0.4–19.8	0.350
G4	50.3	3.0–834.6	0.006			
Lymphangioinvasion						
*L0*	1.0			1.0		
*L1*	2.0	1.2–3.2	0.005	1.3	0.7–2.4	0.394
Venous invasion						
*V0*	1.0			1.0		
*V1*	2.4	1.4–4.2	0.002	2.6	1.4–4.7	0.002
R-stage						0.035
R0	1.0			1.0		
R1	2.3	0.9–5.7	0.072	4.6	1.4–15.2	0.012
Type of operation			0.098			< 0.001
Laparoscopy	1.0			1.0		
Laparotomy	1.9	1.1–3.3	0.032	2.7	1.3–5.7	0.008
Conversion	1.7	0.7–4.1	0.246	13.8	4.8–40.0	< 0.001
Clavien-Dindo						
0–II	1.0			1.0		
III–IV	1.5	0.9–2.4	0.116	2.5	1.4–4.3	0.002

*rCC* right-sided colon cancer, *lCC* left-sided colon cancer, *ASA* American Society of Anesthesiologists, *CEA* carcinoembryonic antigen, *Clavien-Dindo* Clavien-Dindo classification of surgical complications

#### Multivariate analysis

**Fig. 3:**
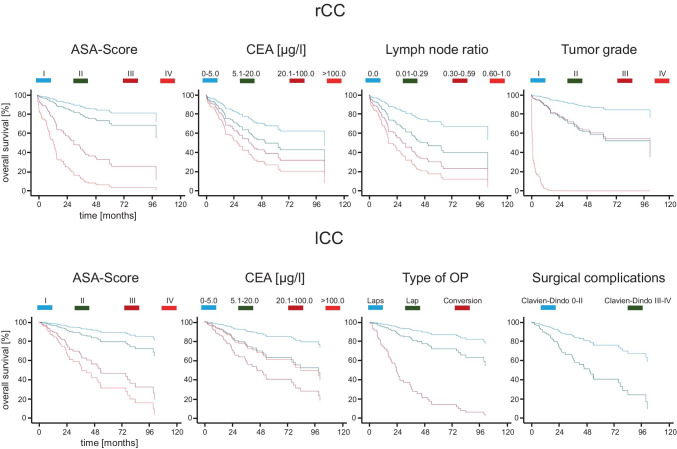
Survival predictors of rCC and lCC: Multivariate analysis of patient- and disease-related parameters disclosed in each group (rCC and lCC) 4 predictors that significantly impacted the overall survival rates. rCC, right-sided colon cancer; lCC, left-sided colon cancer; ASA, American Society of Anesthesiologists; CEA, carcinoembryonic antigen; Laps, laparoscopy; Lap, laparotomy

Parameters with a *p*-value < 0.05 in the univariate analysis were finally applied to a multivariate Cox proportional hazards regression model to evaluate predictors in a cumulative setting. Thus, based on the included factors, we could reveal for each group four patient- and disease-related predictors with distinct hazards that significantly impacted the overall survival rates (Fig. 3 and Table 4). Identical to the univariate analysis, advanced ASA score and elevated CEA blood levels reduced the survival rates in both groups (Fig. 3). However, in the rCC cohort, poor histological grade (G4) (HR: 120.6; CI 95%: 6.7–2179.6, *p* = 0.001) and increased lymph node ratio of 0.6–1.0 (HR: 5.3; CI 95%: 1.7–16.1, *p* = 0.003) were additional prognostic factors associated with a diminished outcome, while type of operation (conversion to laparotomy: HR: 14.1; CI 95%: 4.0–49.0, *p* < 0.001) and grade of surgical complications (Clavien-Dindo III–IV: HR: 2.9; CI 95%: 1.5–5.5, *p* = 0.001) specifically increased the overall mortality risk in lCC patients (Table 4).

**Table 4:** Multivariate analysis of survival predictors of rCC and lCC

rCC	Hazard-ratio	CI 95%	*p*-value
ASA-Score			< 0.001
I	1.0		
II	1.8	0.4–7.9	0.428
III	6.5	1.5–28.0	0.013
IV	16.0	2.1–123.5	0.008
CEA (µg/l)			0.031
0–5.0	1.0		
5.1–20.0	1.8	0.9–3.4	0.081
20.1–100.0	2.4	1.1–5.4	0.034
> 100.0	3.3	1.2–9.0	0.017
Lymph node ratio			0.004
0.0	1.0		
0.01–0.29	2.3	1.3–4.1	0.006
0.30–0.59	3.6	1.5–8.7	0.004
0.60–1.0	5.3	1.7–16.1	0.003
Grade			0.007
G1	1.0		
G2	3.8	0.5–28.5	0.189
G3	3.6	0.5–28.2	0.223
G4	120.6	6.7–2179.6	0.001

lCC	Hazard-ratio	CI 95%	*p-value*
ASA-Score			0.002
I	1.0		
II	2.0	0.3–15.7	0.512
III	6.2	0.8–47.2	0.080
IV	8.9	0.9–91.9	0.068
CEA (µg/l)			0.001
0–5.0	1.0		
5.1–20.0	2.8	1.2–6.2	0.013
20.1–100.0	5.4	2.4–12.4	< 0.001
> 100.0	3.0	0.9–10.0	0.075
Type of operation			< 0.001
Laparoscopy	1.0		
Laparotomy	2.3	1.0–5.5	0.053
Conversion	14.1	4.0–49.0	< 0.001
Clavien-Dindo			
0–II	1.0		
III–IV	2.9	1.5–5.5	0.001

*rCC* right-sided colon cancer, *lCC* left-sided colon cancer, *ASA* American Society of Anesthesiologists, *CEA* carcinoembryonic antigen, *Clavien-Dindo* Clavien-Dindo classification of surgical complications

## Discussion

In the present retrospective single-center study, we analyzed an unselected population of 417 patients that were diagnosed with and treated for CC of any stage. This inclusion criterion was used to provide a realistic representation of a CC patient cohort present at most surgical centers and stands therefore in contrast to previous studies analyzing CC patients’ stage I–III [16, 17]. After undergoing a curative intended oncological resection, our group revealed a 5-y overall survival rate of 62.0% what concurs with broadly accepted outcome findings reviewed by Brenner and colleagues 2014 [22]. The mean (66.9 ± 0.7 years) and median (70 years, 17–98 years) age at the time of diagnosis displayed a similar accordance with the current literature what highlighted our study cohort as a representative demographic population of CC patients [14, 22, 23]. However, sex distribution has shown those heterogenous results that have already been described in previous surveys with a predominant portion of male patients (male: 59.2%; female: 40.8%) present in our study cohort [15, 24]. To disclose potential differences between a right-and left-sided tumor location with respect to clinicopathological characteristics and survival rates, we next examined both groups separately. Thus, we could determine distinct site-specific tumor properties. Compared to lCC patients, rCC patients were predominantly female and displayed an advanced pN-stage (N2b). Furthermore, right-sided carcinoma showed more often a poorer histological grade, were more frequently of a mucinous type, and revealed a high proportion of microsatellite instability what coincides with previous reports [13, 25]. In fact, several studies have already proven genetical and molecular differences between these two tumor locations what substantiate the concept of distinct tumor entities [10]. However, analyzing the survival rates showed no difference of the overall survival between rCC and lCC patients (rCC: 2-y/5-y: 73.0%/ 58.0%; lCC: 2-y/5-y: 77.0%/65.0%) what confirmed the findings of Weiss and colleagues 2011 [24] claiming that tumor location, adjusted for all cancer stages, has no significant impact on mortality. However, these findings conflict with a majority of clinical studies that revealed significant, laterality based, outcome differences associated with a better or worse prognosis [15, 16, 26–29]. Though, observing those trials in detail disclosed essential differences of the tumor locations and stages included in further analyses. (1) Tumors of the transverse colon were sometimes discarded when comparing rCC with lCC patients [16] and (2) many studies were distinguished by a stage-specific study population primary including patients with CC stage I–III, but almost solely excluding stage IV cancer patients or considering them separately [15, 24, 29]. This fact could likely explain the reduced 5-y survival rates of rCC and lCC patients, assessed in the present study, compared to Warschkow and colleagues [16], as CC stage IV patients constituted 20.9% of our total cohort. Comparable to the reduced overall survival, the relatively high proportion of peritoneal metastatic lesions in our study cohort (25.0%) could similarly be ascribed to an increased proportion of pT4 tumors (18.5%) in comparison to previous studies [16, 24] as pT4 status was revealed as an independent predictor of peritoneal recurrence [30]. Otherwise, including CC patients regardless of their stage is indispensable to represent a faithful population present at most surgical centers. Additional to the overall survival rates, we determined the recurrence-free survival of rCC and lCC stage I–III patients which also remained without a significant difference, similar to the findings of Derwinger and Gustavsson 2011 [28].

Even though we could not detect any significant differences of the survival rates among rCC and lCC patients, we still identified numerous outcome predictors that differentially affected the mortality in both groups (Tables 2, 3, and 4 and Fig. 3). ASA score and CEA blood levels showed a significant impact on the overall survival rates in both groups, emphasizing the major influence of comorbidities and tumor load on CC mortality. Moreover, tumor stage altered the overall survival in rCC and lCC what stands in good accordance with the results from Meguid and colleagues 2008 [14]. In fact, an advanced disease at the date of operation due to metastasis, number of affected lymph nodes, or tumor size increases the risk of remaining tumor cells and consequently disease progression with a worse overall outcome. Despite several mortality predictors that were equally found in both groups, we additionally revealed distinct disease- and patient-related parameters that exclusively affected the survival of either rCC or lCC patients. Thus, we could identify a significant impact of an advanced pN-stage and an increased lymph node ratio on mortality in rCC patients. The important role of an extensive lymphadenectomy in rCC could be explained by a congenital increased lymphovascular supply of the right colon and a more aggressive tumor stage at the date of operation. However, it is still unclear, if this observation was caused by an inherent more aggressive tumor biology of rCC or simply the result of a delayed diagnosis leading to an advanced disease with poorer differentiation and increased pN-stage in those patients. Moreover, it remains unsolved, if mucinous carcinoma, frequently observed in rCC, is accompanied with a more severe tumor characteristic [31, 32]. In lCC patients, in contrast, we could detect age and sex as independent predictors that affected the mortality risk, with a worse prognosis for the male sex and patients ≥ 65 years. Furthermore, surgical-related predictors like type of operation and postoperative complications disclosed a worse outcome in those patients who underwent laparotomy or conversion to a laparotomy compared to a primary laparoscopic procedure and who developed severe complications (Clavien-Dindo III–IV). This might reflect adverse intraoperative and perioperative circumstances provoked by a potentially increased likelihood of preoperative complications that are mainly caused by stenosing distal tumors with a consequently increased risk of more severe surgical complications [33, 34]. Finally, by using a multivariate analysis, we assessed the cumulative effect of the investigated covariates on CC mortality according to its location. Thus, beside a general reduction of predictors that significantly influenced the overall survival, we could still identify four parameters in each group. Identical to the abovementioned univariate analysis, ASA score and CEA blood levels remained important survival predictors for both cohorts with the highest mortality risk for ASA IV patients and those presenting markedly increased preoperative CEA-values. Further prognostic factors of rCC were the histological grade and the lymph node ratio what stands in good accordance with the results described by Chapuis and colleagues 1985 [35] for CRC grade and Prandi et al. 2002 [36], as they proved a positive correlation between the number of resected lymph nodes and the overall survival in CC patients. In the lCC cohort, in contrast, the type of operation (conversion to laparotomy) and severe surgical complications (Clavien-Dindo III–IV) were significantly correlated with an increased mortality. As stenosing CC with its complications is mainly found in the left part of the colon [33], the consequent higher risk of preoperative adverse circumstances could explain the observed effect due to subsequently reduced perioperative and intraoperative conditions leading to a revision of the initially intended surgical procedure. The likelihood of severe postoperative complications is then similar affected as a consequence of the perioperative and intraoperative circumstances. In the end, an important limitation of this study was a reduced follow-up period. On the one hand, this fact could have been caused by an incomplete follow-up data acquisition when therapy was sometimes proceeded elsewhere additionally leading to prematurely censored events in the survival analysis. A further reason was potentially the inclusion of patients with CC stage IV who generally show reduced survival times [29] and consequently short follow-up periods. A second limitation was the single-center characteristic. Although including patients with CC stage I–IV, high-volume hospitals with special surgical expertise tend to treat predominantly patients of an advanced disease stage with worse perioperative conditions what could have biased the overall outcome findings of this trial. Furthermore, we could not fully determine the molecular properties of rCC and lCC. Although testing for microsatellite instability in 65.7% of our patients, further molecular markers like BRAF and KRAS were not determined, but have been shown to display a strong association with survival in CC [37–39]. Finally, as disease progression and mortality are positively correlated with the number of circulating tumor cells, the fact of including patients with pM1a-c status could have impeded this observation due to an increased tumor load with a consequently higher probability of remaining tumor cells despite a curative intended surgical approach.

## Conclusion

rCC and lCC stage I–IV patients showed no significant difference in their overall survival rates; hence, tumor location could not be classified as an independent mortality predictor in the present heterogenous and unselected study cohort. However, several group-specific patient- and disease-related parameters could be identified to alter the mortality risk like ASA score, CEA blood level, histological grade, lymph node ratio, surgical procedure, and severity of surgical complications what might support the concept of locally separated tumor entities. Additionally, the fact of revealing distinct risk factors could have direct ramifications on future treatment strategies and could pave the way for prospective randomized trials. Beside a closed follow-up for all patients with the abovementioned risk factors, ASA IV patients, in particular, could probably benefit from specific prehabilitation programs whose effect on postoperative complications has been previously studied [40, 41]. Prehabilitation combined with a multidisciplinary preoperative treatment strategy might also help to select the optimal surgical procedure and could avoid the number of conversions. Furthermore, the effect of a more stringent and escalated (neo)-adjuvant chemotherapy regimen on the overall survival of patients with G4 tumors and markedly increased CEA values could be explored in future studies. Finally, a systematic lymphadenectomy using intraoperative lymph node mapping techniques [42] might help to reduce the overall mortality risk especially in the rCC cohort.

## Data Availability

The dataset of the current study is available from the corresponding author on reasonable request.

## Code availability

All analyses presented in tables and figures were performed with IBM SPSS Statistics 27.0 (IBM Corp. Released 2020. IBM SPSS Statistics for Windows, Version 27.0. Armonk, NY: IBM Corp). A custom code was not used in this study.
